# The regulation of toll-like receptor 2 by miR-143 suppresses the invasion and migration of a subset of human colorectal carcinoma cells

**DOI:** 10.1186/1476-4598-12-77

**Published:** 2013-07-17

**Authors:** Haiyan Guo, Ying Chen, Xiaobo Hu, Guanxiang Qian, Shengfang Ge, Jianjun Zhang

**Affiliations:** 1 Department of Clinical Laboratory, Shanghai Third People’s Hospital, School of medicine, Shanghai Jiao Tong University, Shanghai 200019, PR China; 2 Department of Oral & Maxillofacial-Head Neck Oncology, Ninth People’s Hospital, Shanghai Jiao Tong University School of Medicine, Shanghai 200011, PR China; 3 Department of Ophthalmology, Ninth People’s Hospital, Shanghai Jiao Tong University School of Medicine, Shanghai, PR China; 4 Department of Biochemistry and Molecular Biology, Shanghai Jiao Tong University School of Medicine, Shanghai, PR China

**Keywords:** miR-143, Toll-like receptor 2, Colorectal carcinoma, Invasion, Migration

## Abstract

**Background:**

The Toll-like receptor 2 (TLR2)-driven tissue response may promote neoangiogenesis and tumour growth by mechanisms that are poorly understood.

**Methods:**

We investigated the expression levels of TLR2 and associated-miRNAs in colorectal carcinoma (CRC) tissues and cell lines using real-time PCR, northern blotting and western blotting. Survival curver was generated by Log-Rank test and the role of TLR2 signalling in tumour invasion and migration was determined by transwell analysis kits.

**Results:**

We observed that the tissues from CRC patients express relatively high levels of TLR2. Targeting TLR2 markedly reduces the invasion and migration of CRC cells. We also found that miR-143, a putative tumour suppressor that is down-regulated in CRC tissues, reduces the invasion and migration of CRC cells primarily via TLR2. Utilising a xenograft mouse model, we demonstrated that re-expression of miR-143 inhibits CRC cell colonisation in vivo.

**Conclusion:**

miR-143 blocks the TLR2 signalling pathway in human CRC cells. This knowledge may pave the way for new clinical applications utilising miR-143 mimics in the treatment of patients with CRC.

## Introduction

Colorectal carcinoma (CRC) is a commonly diagnosed cancer in males and females. It is estimated that over 1.2 million new cancer diagnoses and 608,700 deaths from colorectal cancer occurred in 2008 [1]. Tumour metastasis is the most common cause of death in patients with CRC, but there are no effective therapies that target the development and progression of metastasis [2]. One therapeutic strategy against tumour invasion and migration of recent interest is immunotherapy using agonists of Toll-like receptors (TLRs) as adjuvants [3-5].

The TLRs are a family of receptors consisting of highly conserved molecules, that recognise the pathogen-associated molecular patterns (PAMP) of microorganisms and viruses and play critical roles in the innate immune response and subsequent induction of the adaptive immune response [6-8]. Moreover, TLRs induce chronic inflammation after being activated by damage-associated molecular patterns (DAMPs) released from injured tissue or tumour tissue [9,10]. Activation of TLR signalling in the steady state maintains tissue architecture. However, in the presence of the deregulated inflammation that occurs during tumourigenesis, the TLR-driven tissue response may promote neoangiogenesis and tumour growth by poorly defined mechanisms [11]. Recent studies have demonstrated that engagement of TLRs increases tumour growth and tumour immune evasion and induces resistance to apoptosis and chemoresistance in some tumour cells [12,13], implying the possibility of TLR-targeted cancer therapy. In lung cancer, it has been shown that engagement of TLR4 and TLR9 induces apoptosis in cancer cells [14]. In ovarian cancer cells, it has been shown that activation of TLR4 signalling results in increased growth and chemoresistance [13]. These findings demonstrate that different tumours express different functional TLRs, and the effects of TLR signalling in cancer cells may vary according to cancer cell type.

A growing body of evidence suggests that the Toll-like receptor 2 (TLR2) in the intestinal epithelium of patients with CRC or inflammatory bowel disease is up-regulated in comparison with healthy individuals [15-17]. In general, stimulation of TLR2 induces a TH2, Treg, or TH17 type of immune response [18,19]. It is clear that TLR2 has a unique position in the regulation of tumour tolerance, cancer progression and metastasis [20].

In this study, we identified a putative tumour suppressor miRNA, miR-143, that is frequently down-regulated in CRC. We demonstrated that miR-143 can directly regulate TLR2 expression in CRC cells. High levels of miR-143 can inhibit tumour invasion and migration in vitro and in vivo by reducing TLR2 activity. Our study provides strong evidence that TLR2 may be targeted by miR-143 to overcome chronic inflammation and down-regulate tumour invasion and migration.

## Materials and methods

### Ethics statement and human colorectal carcinoma tissues

The specimens were collected from patients who underwent surgery at the Shanghai Renji Hospital between January 2004 and January 2010. The mean follow-up time was 92 months (from 41 to 122 months). The protocol was approved by the Shanghai Jiao Tong University School of Medicine Clinical Research Ethics Committee, and the research was performed according to the 1975 Helsinki Declaration provisions. Written informed consent was obtained from all participants involved in the study.

### Cell lines

The colorectal carcinoma cell lines, including LS174T (ATCC number: CL-188™, Dukes’ type B cells), SW480 (ATCC Number: CCL-228™, Dukes’ type B cells), SW620 (ATCC Number: CCL-227™, Dukes’ type C cells) and HCT116 (ATCC number: CCL-247, Dukes’ type D cells), were purchased from the Cell Bank of Type Culture Collection of the Shanghai Institute of Cell Biology, Chinese Academy of Sciences. These colorectal carcinoma cell lines were maintained in RPMI 1640 containing 10% foetal calf serum. Cultures were incubated at 37°C in standard tissue culture incubators.

### Real-time PCR

Total RNA was extracted from cells using TRIzol Reagent (Life Technologies, Gaithersburg, MD, USA). Real-time PCR analyses were performed to detect TLR2 mRNA expression using SYBR Premix Ex Taq (TaKaRa, Dalian, China), and GAPDH was used as an internal control. Real-time PCR was performed under the following conditions: 95°C 10 m, 1 cycle; 95°C 10 s, 55°C 34 s, 40 cycles. The PCR primers were TLR2 forward 5′ GATGC CTACT GGGTG GAG 3′, TLR2 reverse 5′ AAAGA CGGAA ATGGG AGA 3′; GAPDH forward: 5′ TGGGG AAGGT GAAGG TCGG 3′ and GAPDH reverse: 5′ CTGGA AGATG GTGAT GGGA 3′.

The MiRcute miRNA qPCR detection kit (TIANGEN, Beijing, China) was used to quantify the expression levels of mature miR-143 according to the protocol provided, and GAPDH was used as an internal control. The primer sequence used was the miR-143 specific primer 5′ TGAGA TGAAG CACTG TAGCT C 3′. Real-time PCR was performed under the following conditions: 95°C 1 m, 1 cycle; 95°C 10 s, 65°C 40 s, 40 cycles.

For all results obtained by real-time PCR methods, we used the delta delta CT method to calculate the fold change in gene expression between different groups. We used a housekeeping gene (GAPDH) for endogenous normalisation. The amount of target (TLR2/miR-143), normalised to the endogenous housekeeping gene GAPDH and relative to a reference sample, is given by the following equation: amount of target =2^-△△CT^.

### Vector constructs

The full length wild type 3′UTR (UTR-WT) of the TLR2 mRNA containing the putative miR-143 binding sites was amplified by PCR and cloned into the Xba1 site of the pGL3 control vector (Promega, Madison, WI, USA). The amplified PCR primers used were TLR2 UTR-WT forward: 5′ GATGC CTACT GGGTG GAG 3′, and TLR2 UTR-WT reverse 5′ AATAC TTTGC CTTGT TGC 3′. The mutant 3′UTR of TLR2 (UTR-MUT), which carried a mutation in the complementary site for the seed region of miR-143, was generated from the UTR-WT plasmid by overlap-extension PCR. The TLR2 transcript with a deletion mutation for 3′UTR (TLR2) was amplified and cloned into the pcDNA3.0 vector. The amplified PCR primers used were TLR2 CDS forward: 5′ CTGGA CAATG CCACA TAC 3′, and TLR2 CDS reverse: 5′ AAGAT CCCAA CTAGA CAAA 3′.

### Oligonucleotide transfection

S transfectants over-expressing miR-143 were generated by lentiviral transduction using a pMIRNA1 plasmid carrying miR-143 (System Biosciences, Mountain View, CA). Mimics and inhibitors of miR-143 were purchased from Dharmacon (Lafayette, CO, US). Transient transfections of miR-143 mimics/inhibitors in cancer cells were performed using Lipofectamine™ 2000 (Invitrogen, Carlsbad, CA, USA) following the manufacturer’s protocol. The final concentration of miR-143 mimics/inhibitors in the transfection system was 100 nM. As a positive control, the mature sequence of has-miR-19a is 5′-UGUGC AAAUC UAUGC AAAAC UGA-3′. The final concentration of miR-19a mimics in the transfection system was 100 nM.

For the TLR2 knockdown, the sequences of the siRNA were: 5′-GGGCA GUCUU GAACA UUUAU U-3′ and 5′-UAAAU GUUCA AGACU GCCCU U-3′. All of the RNAi oligoribonucleotides were purchased from Genepharma (Shanghai, China). Transfection was performed with Lipofectamine™ 2000 following the manufacturer’s protocol. A final concentration of 100 nM of TLR2 siRNA and the negative controls was used for each transfection. Twenty-four hours after transfection, the biological behaviour of the cancer cells was observed.

### Luciferase assay

SW620 cells were seeded in 24-well plates and co-transfected with 100 nM of miR-143 mimics, 100 ng/ml UTR-WT or UTR-MUT luciferase reporter construct, and 10 ng/ml pRL-CMV Renilla luciferase reporter using lipofectamine 2000. Cells were collected 24 h after transfection, and luciferase activity was measured with a dual-luciferase reporter assay (Promega, Madison, WI, USA). The luciferase activity was normalised to the Renilla luciferase activity.

### Northern blotting

We extracted RNA with the mirVana™ miRNA Isolation kit (Ambion, Austin, TX) using the microRNA enrichment protocol. Mature miR-143 was measured by northern blotting using a Northern Max-Gly Kit (Ambion, Austin, TX). Briefly, after RNA electrophoresis, the transferred membrane was prehybridised with ULTRAhyb and detected with a miR-143-specific oligonucleotide probe (5′ GAGCT ACTGT GCTTC ATCTC A 3′) labelled with digoxigenin-ddUTP using a DIG Oligonucleotide 3′-End Labeling Kit (Roche Diagnostics, Indianapolis, IN). U2snRNA was used as an internal control. The U2snRNA-specific oligonucleotide probe used was 5′ TCGGA TAGAG GACGT ATCAG ATATT AAA 3′.

### Western blot

Proteins were separated on a 10% SDS-PAGE gel and then transferred to a PVDF membrane. The membrane was incubated with a rabbit TLR2 polyclonal antibody (1:1000, Proteintech, Chicago, IL, USA). The secondary antibodies were labelled with IRDyes. The signals were observed using an Odyssey Infrared Imaging System (LI-COR Biosciences, Lincoln, NE, USA).

### Growth assay

The proliferation assay was performed with the Cell Counting Kit-8 (Dojindo, Kumamoto, Japan) according to the manufacturer’s instructions. Before the addition of CCK-8, the cells were washed with warm culture media by spinning the plate at 500 rpm for 3 m and then discarding the supernatant.

### Cell migration and invasion assays

Transwell migration and invasion assays were performed as described previously [21]. The cell invasion assay was performed with matrigel (BD Biosciences, Sparks, MD) coated on the upper surface of a Transwell chamber (Corning, Lowell, MA). The cell migration assay was performed in a Transwell chamber untreated with matrigel. The cells that had migrated or invaded through the membrane were fixed with 4% paraformaldehyde and stained with Coomassie brilliant blue methanol. Photographs of 3 randomly selected fields of the fixed cells were taken, and the cells were counted.

### Mouse experiments

Aliquots of 1 ×10^6^ stable miR-143 or miR-NC over-expressing SW620 cells were injected into immunodeficient mice and evaluated for lung colonisation capacity in tail-vein assays. Lung colonisation was measured using bioluminescence at 1, 2, 3, 4, 5 and 6 weeks after injection.

### Statistical analyses

The data are shown as the mean ± SD. Multiple group comparisons were performed by one-way ANOVA, and survival curver was generated by Log-Rank test followed by the SPSS procedure for comparison of means. A *p < 0.05* was considered to be significant.

## Results

### TLR2 is highly expressed in human CRC tissues and cell lines

**Figure 1:**
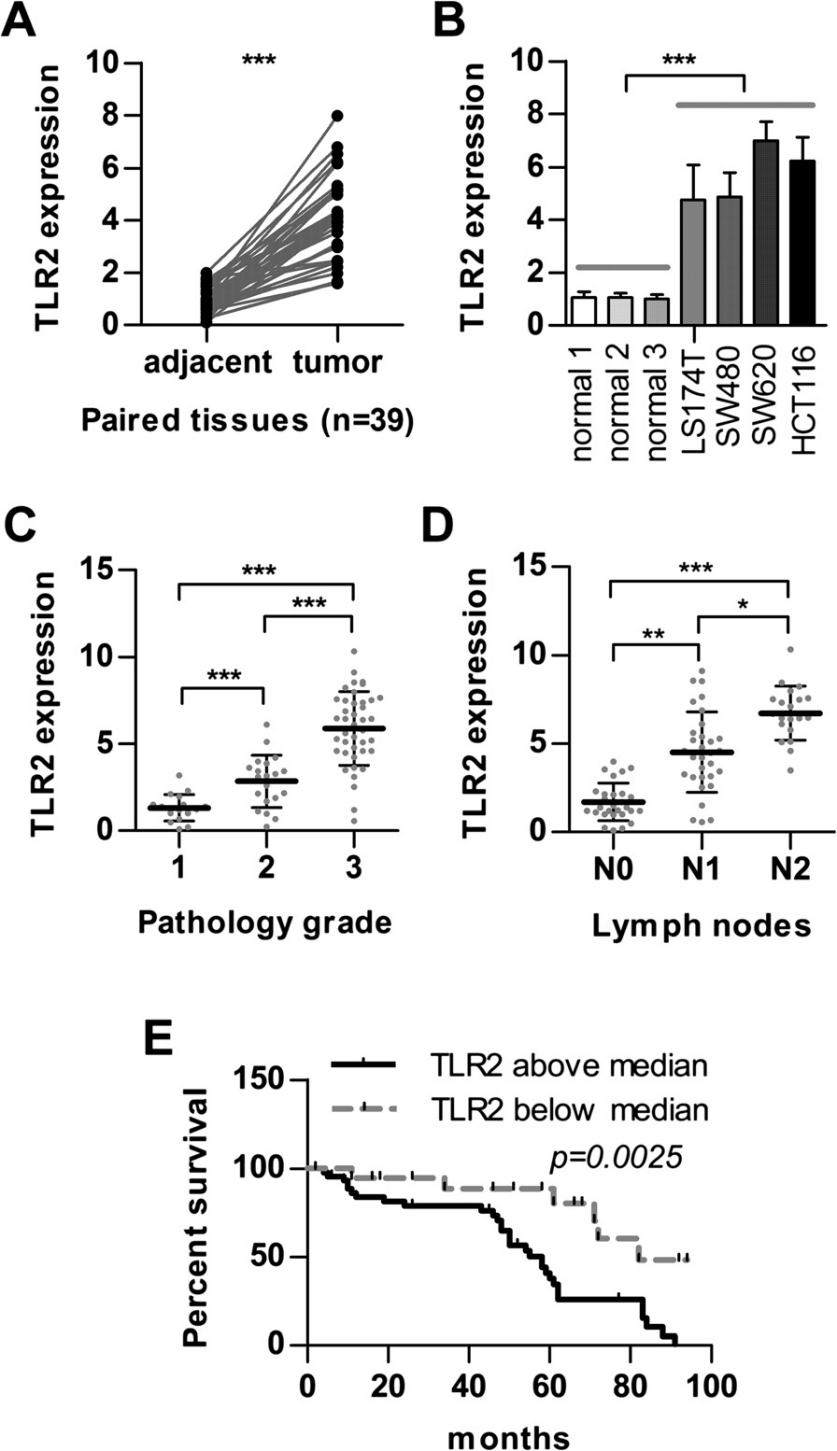
**High TLR2 levels are associated with malignant transformation and lower overall survival in CRC patients. **(**A**) Expression of TLR2 in 39 pairs of tumour samples and matched adjacent noncancerous tissues was measured by real-time PCR. (**B**) Expression of TLR2 was determined by real-time PCR in 3 normal colon epithelial cells and 4 CRC cell lines (SW480, LS174T, SW620, and HCT116). (**C**) Expression of TLR2 was determined in 79 CRC tissues (grade1 n = 18; grade2 n = 20; grade3 n = 41) by real-time PCR. The statistical method analysed the correlation between the expression of TLR2 and tumour pathological grade. (**D**) Expression of TLR2 was checked in 79 CRC tissues (N0 n = 28; N1 n = 31; N2 n = 20) by real-time PCR. The statistical method analysed the correlation between the expression of TLR2 and tumour lymph node metastasis. (**E**) Lower overall survival of patients with high TLR2 levels (above median; *n* = 37) compared to patients with low TLR2 levels (below median; *n* = 42). (***p < 0.001; **p < 0.01; *p < 0.05).

To determine the potential roles of TLRs in the regulation of tumourigenesis, we first examined the expression of TLRs in CRC. Using real-time PCR and immunohistochemistry analysis, we analysed TLR2 expression in 39 paired samples (tumour and adjacent noncancerous tissues from the same patient) and found that TLR2 expression is significantly up-regulated in CRC tissues (Figure 1A and Additional file 1: Figure S1; ***p < 0.001). We observed that TLR2 expression was significantly up-regulated in 4 CRC cell lines compared with normal epithelial tissues (Figure 1B; ***p < 0.001). Additionally, we detected higher TLR2 expression in poorly differentiated tumour cells (SW620 and HCT116) compared with well-differentiated tumour cells (SW480 and LS174T). The TLR2 expression profile of human CRC tissues revealed that high levels of TLR2 correlated with more advanced pathology grades (1, 2, 3) and lymph node metastasis (N0, N1, N2) (Figures 1C and 1D; ***p < 0.001; **p < 0.01; *p < 0.05), suggesting an association between TLR2 expression and tumour progression. Accordingly, TLR2 levels above the median correlate with lower overall survival of patients with CRC (Figure 1E; *p = 0.0025*). The characteristics of the patients with colorectal carcinoma are listed in Table 1.

**Table 1:** Characteristics of the 79 colorectal carcinoma patients

**Variables**	**Number (%)**
Gender (male/female)	39 (49.4.)/40 (50.6)
Age (mean±SD)	53.4 ± 15.2
Maximum size (<5 cm/≥5 cm)	38 (48.1)/41 (51.9)
Stage (I/II/III/IV)	7 (8.9)/21 (26.6)/32 (40.5)/19 (24.0)
Pathology grade (1/2/3)	18 (22.8)/20 (5.3)/41(51.9)
Lymph nodes (N0/N1/N2)	28 (35.4)/ 31(39.2)/20 (25.3)
Vascular invasion (no/yes)	36 (45.6)/43 (54.4)
Preoperative chemotherapy* (no/yes)	66 (83.5)/13(16.5)
Postoperative chemotherapy* (no/yes)	19 (24.1)/60(75.9)
Mean follow- up time (months)	92 (from 41 to 122).

* Chemotherapy: 5-FU & low-dose Leucovorin (Mayo Clinic regimen/LDLV).

### TLR2 mediates the capacity for invasion and migration in CRC cells

**Figure 2:**
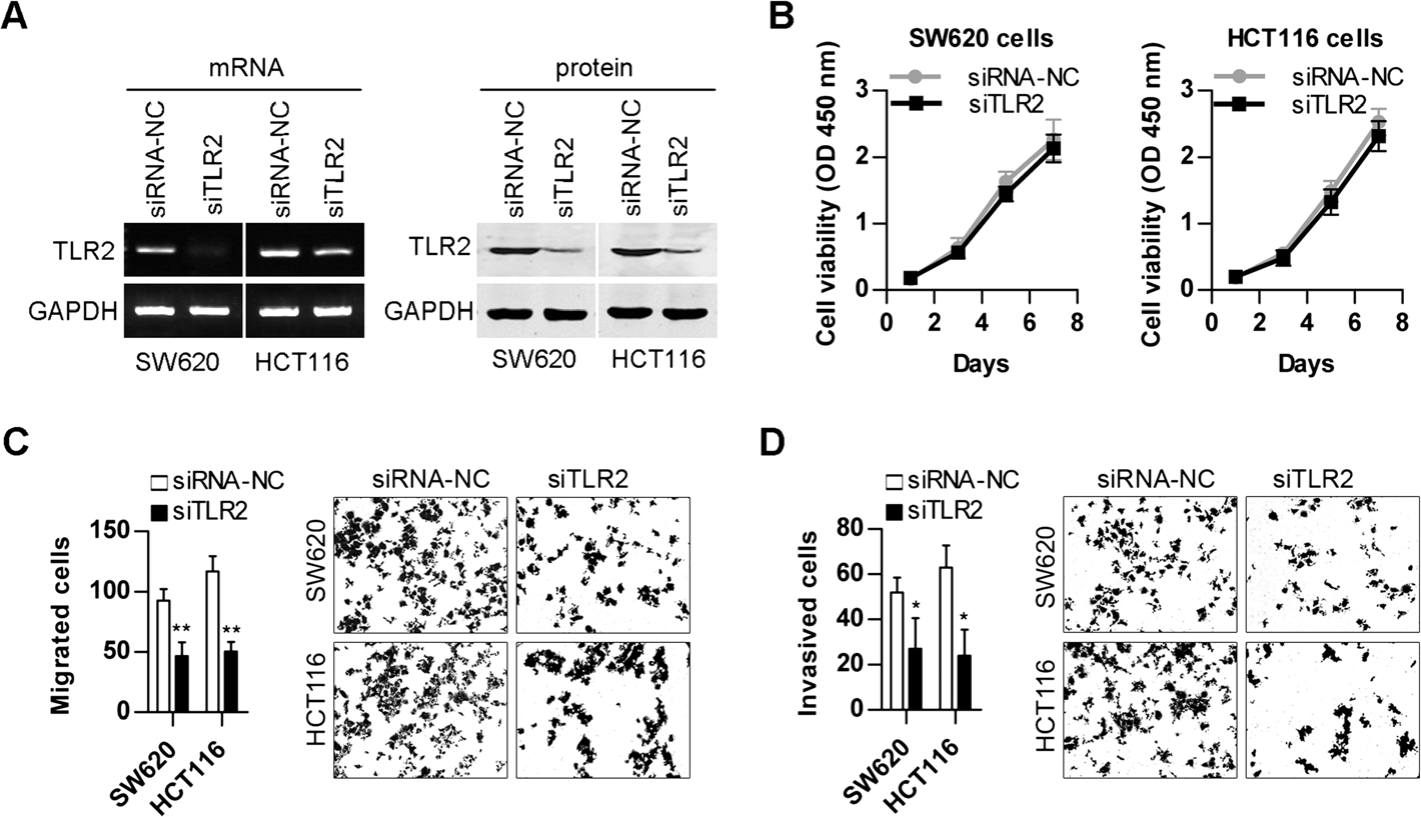
**High TLR2 levels facilitate CRC cell migration and invasion.** (**A**) Expression of TLR2 was measured in SW620 and HCT116 cells treated with siRNA for TLR2 (siTLR2). (**B**) Proliferation was detected using a Cell Counting Kit-8 in SW620 and HCT116 cells treated with siTLR2. (**C**) Migration of SW620 and HCT116 cells treated with siTLR2 was determined using Transwell chambers untreated with matrigel. (**D**) Invasion of SW620 and HCT116 cells treated with siTLR2 was determined using matrigel-treated Transwell chambers. (**p < 0.01; *p < 0.05).

We examined the role of TLR2 in CRC cell invasion, migration, and growth. To examine the down-regulating effect of siRNA on TLR2 expression, we performed a qRT-PCR analysis (Figure 2A and Additional file 2: Figure S2A; left panel) and a western blot analysis (Figure 2A and Additional file 2: Figure S2B; right panel) 48 h after transfecting SW620 and HCT116 cells with siRNA for TLR2 (siTLR2). As shown in Figure 2A, compared to the negative control group (siRNA-NC), ectopic expression of siTLR2 significantly decreased the expression of TLR2.

Cell migration and invasion are crucial steps in the physiopathology of many diseases, including cancer. The migration and invasion of tumour cells into surrounding tissues is a pernicious progression, resulting in the development of distant metastases [22]. In comparison with the negative control (siRNA-NC), TLR2 knockdown significantly inhibited the invasive and migratory activities of SW620 and HCT116 cells (Figures 2C and 2D, **p < 0.01; *p < 0.05). However, TLR2 knockdown did not affect the growth of SW620 and HCT116 cells (Figure 2B; *p > 0.05*). Transfection with the TLR2 expression vector (TLR2) significantly increased cell invasion and migration in normal colon epithelial cells (Additional file 2: Figures S2C and S2D). Additionally, TLR2 was over-expressed in several human tumour cell lines, such as gastric carcinoma cells and melanoma cells [23,24]. Our results showed that knockdown of TLR2 markedly reduced the invasive capacity of highly metastatic cancer cell lines, including human lung adenocarcinoma A549 cells and human breast cancer MDA-MB-231 cells (data not shown).

### TLR2 expression correlates with miR-143 in CRC tissues and cells

MicroRNAs (miRNAs) have emerged as important regulators of cell signalling mediators via interfering with mRNA stability or translation [25,26]. To identify potential miRNAs that are involved in TLR2 signalling, we searched for miRNAs targeting TLR2 using microrna.org (http://www.microrna.org/microrna/getGeneForm.do), TargetScan (http://www.targetscan.org/), and Microcosm Targets (http://www.ebi.ac.uk/enright-srv/microcosm/htdocs/targets/v5/) software. By comparing the results of the predictive algorithms, we identified miR-101, miR-143, miR-154, and miR-340 as the candidate TLR2-targeting miRNAs. The predicted binding of miRNAs with the 3′UTR of TLR2 is illustrated in Additional file 3: Figure S3D.

**Figure 3:**
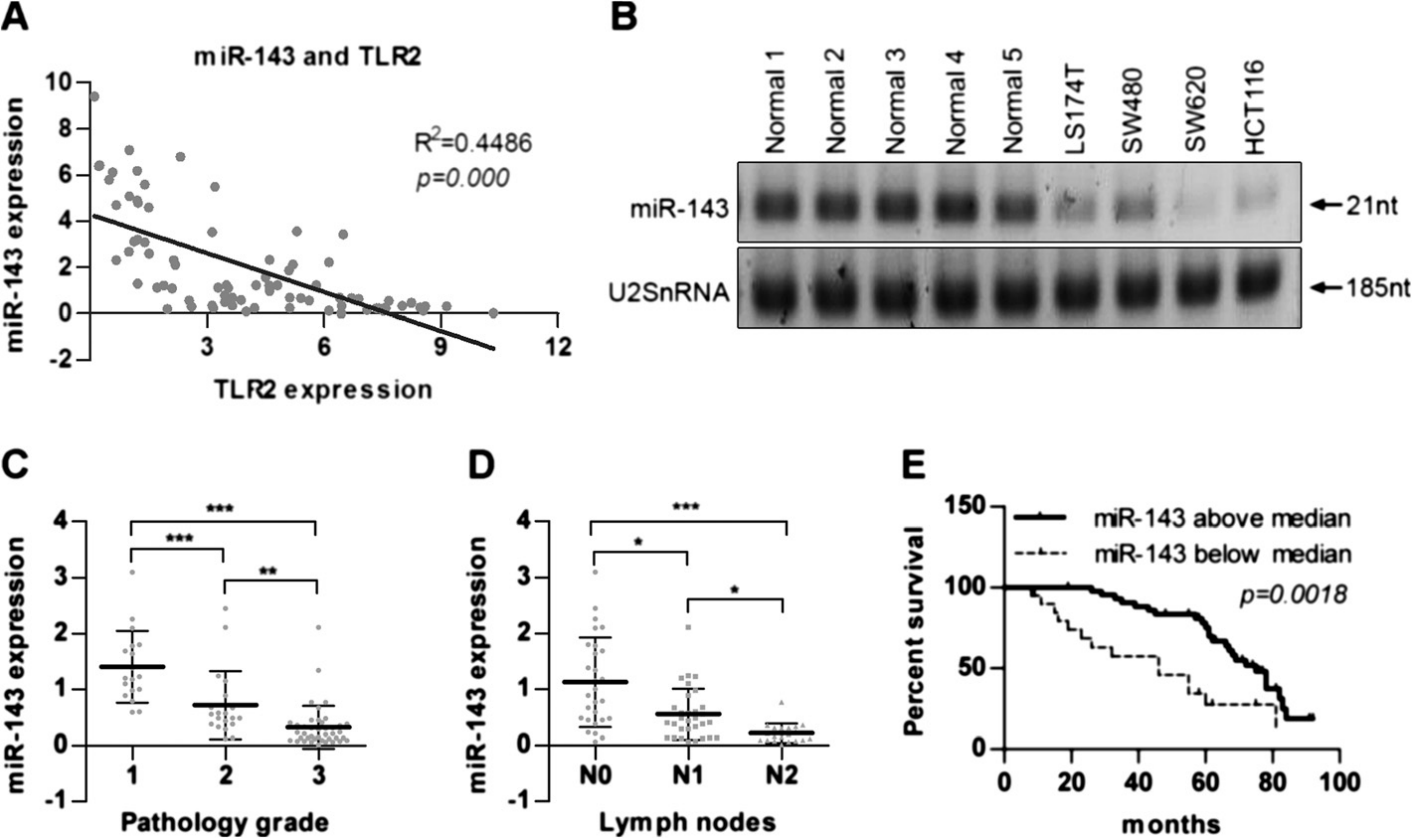
**Expression levels of TLR2 are inversely correlated with miR-143 in CRC tissues and cell lines. **(**A**) Expression of miR-143 was determined using real-time PCR in 79 CRC tissue samples, and the correlation of TLR2 expression levels with miR-143 in CRC tissues was evaluated using nonparametric tests (R^2^ = 0.4486; *p = 0.000*). (**B**) Expression of miR-143 was determined by northern blotting in 5 normal colon epithelial cells and 4 CRC cell lines (SW480, LS174T, SW620, and HCT116). (**C**) Expression of miR-143 was determined in 79 CRC tissues (grade1 n = 18; grade2 n = 20; grade3 n = 41) by real-time PCR. The statistical method analysed the correlation between the expression of miR-143 and tumour pathological grade. (**D**) Expression of miR-143 was determined in 79 CRC tissues (N0 n = 28; N1 n = 31; N2 n = 20) by real-time PCR. The statistical method analysed the correlation between the expression of miR-143 and tumour lymph node metastasis. (**E**) Higher overall survival of patients with high miR-143 levels (above median; n = 38) compared to patients with low miR-143 levels (below median; n = 41). (***p < 0.001; **p < 0.01; *p < 0.05).

We detected the expression of TLR2 and mature miR-143 in the same clinical samples using real-time PCR (N = 79). Using nonparametric tests, we found a significant inverse correlation between TLR2 expression and miR-143 expression in CRC tissues (R^2^ = 0.4486, *p = 0.000* for TLR2 and miR-143) (Figure 3A; Table 1). We utilised the same tests to determine whether TLR2 is a putative target of miR-101, miR-154, and miR-340 and found no inverse correlation between TLR2 and these miRNAs in human CRC samples (data not shown). In addition, we examined the endogenous miR-143 expression levels by northern blotting in 5 normal colon epithelial tissues and 4 CRC cells. The expression of miR-143 was significantly lower in 4 CRC cells with high TLR2 expression levels (Figure 3B and Additional file 3: Figure S3A), especially in the poorly differentiated tumour cells SW620 and HCT116, showing that miR-143 levels are inversely correlated with the levels of TLR2 expression. The miR-143 profile of human CRC tissues revealed that low levels of miR-143 are correlated with more advanced pathology grades (1, 2, 3) and lymph node metastasis (N0, N1, N2) (Figures 3C and 3D; ***p < 0.001; **p < 0.01; *p < 0.05), suggesting an association between low miR-143 expression and tumour progression. Additionally, miR-143 levels above the median correlate with a higher overall survival of patients with CRC (*p = 0.0018*) (Figure 3E).

### miR-143 directly targets TLR2 and reverses the invasive and migratory phenotype in CRC cells

SW620 and HCT116 cells, which express very little mature miR-143, were transfected with a miRNA mimic (miR-143 mimic) and its inhibitor (Anti-miR-143). The ectopic expression of mature miR-143 was confirmed by real-time PCR assay. As expected, an approximately 10-fold increase in mature miR-143 was detected in the miR-143 mimic transfected cells (Additional file 3: Figure S3B). In contrast, transfection with the miR-143 inhibitor (Anti-miR-143) reduced mature miR-143 by almost 70% in SW620 and HCT116 cells (Additional file 3: Figure S3C).

**Figure 4:**
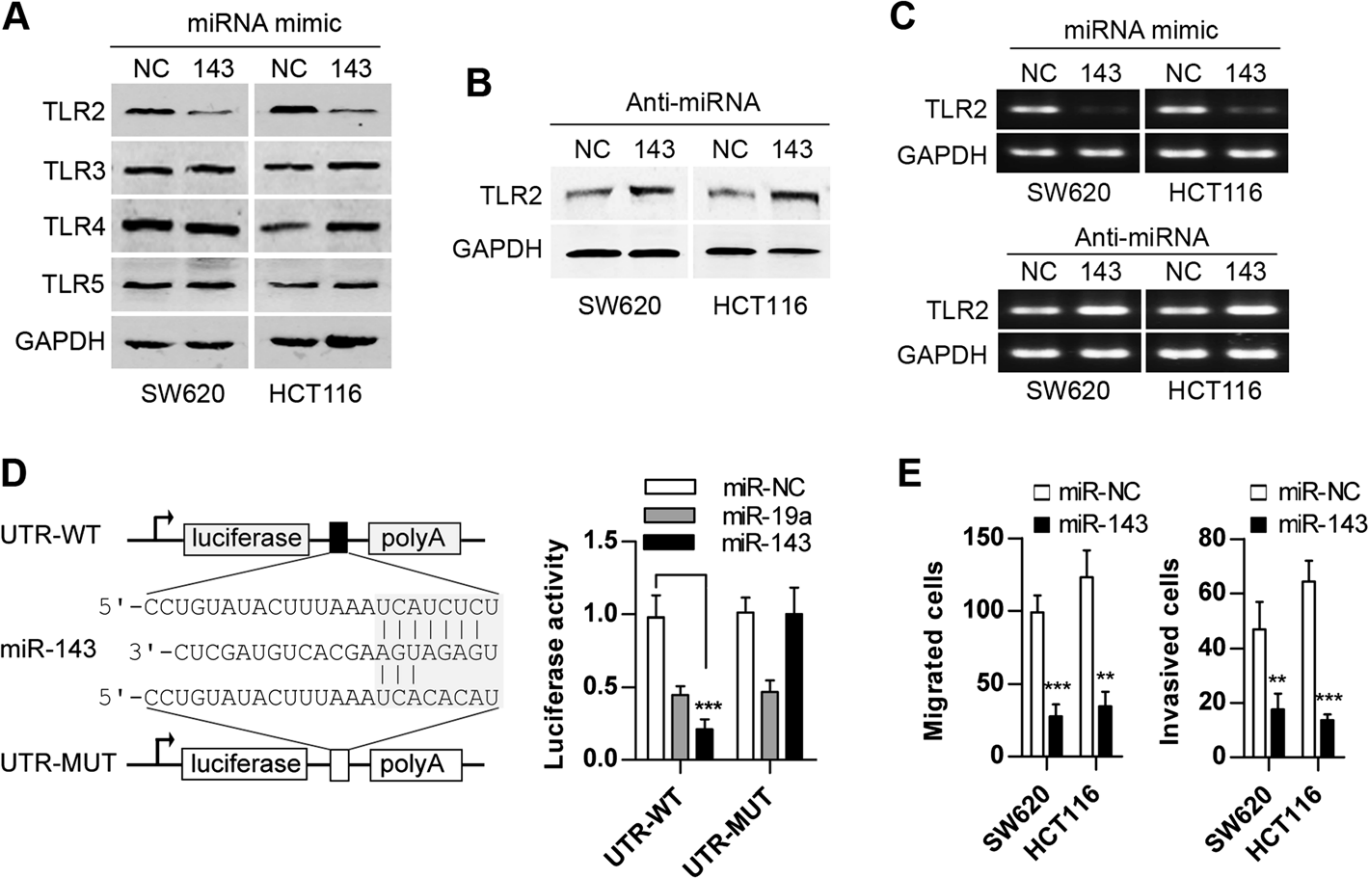
**miR-143 directly targets TLR2.** (**A**) Expression of TLR2, TLR3, TLR4, and TLR5 was measured in SW620 and HCT116 cells transfected with miR-143. NC is a double-stranded RNA and represents the miRNA mimic negative control. (**B**) Expression of TLR2 was measured in SW620 and HCT116 cells transfected with Anti-miR-143. NC is a single-stranded RNA and represents the anti-miRNA negative control. (**C**) The levels of TLR2 mRNA are negatively regulated by miR-143 in SW620 and HCT116 cells. (**D**) The sequence alignment of miR-143 with the putative binding site in the 3*'*UTR of the TLR2 gene. Reporter assay in SW620 cells transfected with luciferase constructs (UTR-WT or UTR-MUT) (mean ± SD). (**E**) Inhibition of migration and invasion of cancer cells by miR-143 was measured using Transwell chambers untreated with matrigel and matrigel-treated Transwell chambers. (WT: wild type; MUT: mutant; **p < 0.01; ***p < 0.001).

To examine the down-regulatory effect of miR-143 on TLR2 expression, we performed a western blot analysis 48 h after transfecting SW620 and HCT116 cells with the miR-143 mimic (100 nM) or Anti-miR-143 (100 nM). Compared to the negative control group (miR-NC mimic), ectopic expression of miR-143 significantly decreased the expression of TLR2 protein levels (Figures 4A and Additional file 4: Figure S4A). This inhibition was abolished by treatment with Anti-miR-143, which is an antagonist for miR-143 (Figures 4B and Additional file 4: Figure S4B). Previous studies have reported that the Toll-like receptor family members TLR3 [27], TLR4 [28], and TLR5 [29] were up-regulated in colorectal carcinoma tissues. Therefore, we examined whether TLR3, TLR4, and TLR5 are also regulated by miR-143 in human CRC. The expression of TLR3, TLR4, and TLR5 protein was not affected by miR-143 in SW620 and HCT116 cells (Figures 4A and Additional file 4: Figure S4A). Using a qRT-PCR assay, we detected miR-143-induced down-regulation of TLR2 mRNA expression in SW620 and HCT116 cells, and a blockage of endogenous miR-143 up-regulated expression of TLR2 mRNA in SW620 and HCT116 cells (Figures 4C and Additional file 4: Figure S4C). These results suggest that miR-143 targets TLR2 by playing a role in mRNA cleavage.

To further confirm that TLR2 is a direct target of miR-143, a firefly luciferase reporter assay system was used. We amplified the complete 3′UTR of TLR2 mRNA and cloned it into the 3′UTR of the firefly luciferase gene in the reporter vector UTR-WT. Using the UTR-WT vector as a template, we constructed a UTR-MUT vector containing the 3′UTR of TLR2 with point mutations, as shown in Figure 4D. In addition, miR-19a was used as a positive control, as it has been reported to target TLR2 in Rheumatoid Fibroblast-like Synoviocytes [30]. The UTR-WT or UTR-MUT vector was co-transfected with miR-143 mimic (miR-143), miR-19a mimic (miR-19a) or miRNA mimic control (miR-NC) into SW620 cells. As expected, co-transfection with miR-19a dramatically attenuated UTR-WT luciferase activity. Interestingly, the UTR-WT luciferase activity in miR-143 co-transfected SW620 cells was significantly lower than in the cells that were co-transfected with the miR-NC, whereas mutation of the miR-143 recognition site rescued the luciferase activity (Figure 4D). These results collectively suggest a direct and specific inhibition of the 3′UTR of TLR2 by miR-143 in CRC cells.

TLR2 has been shown to promote invasion and migration in CRC cells (Figures 2C, 2D, Additional file 2: Figure S2C and S2D). This finding suggests that re-expression of miR-143 may lead to a reversal of the invasive and migratory phenotypes in CRC cells. To test our hypothesis, the effect of ectopic miR-143 expression on cell invasion and migration was investigated in SW620 and HCT116 cells. Transfection with miR-143 significantly decreased cell invasion and migration in SW620 cells and HCT116 cells (Figure 4E). Repression of TLR2 by miR-143 can partially recapitulate the results of silencing TLR2 using RNA interference in SW620 and HCT116 cells (Figures 2C and 2D). Inversely, miR-143 knockdown significantly increased the invasive and migratory activities of normal colon epithelial cells (Additional file 4: Figures S4D and S4E). These results indicate that re-expression of miR-143 dramatically decreases cell invasion and migration in TLR2-expressing CRC cells.

### TLR2 mediates the miR-143-induced resistance to invasion and migration in vitro and in vivo

**Figure 5:**
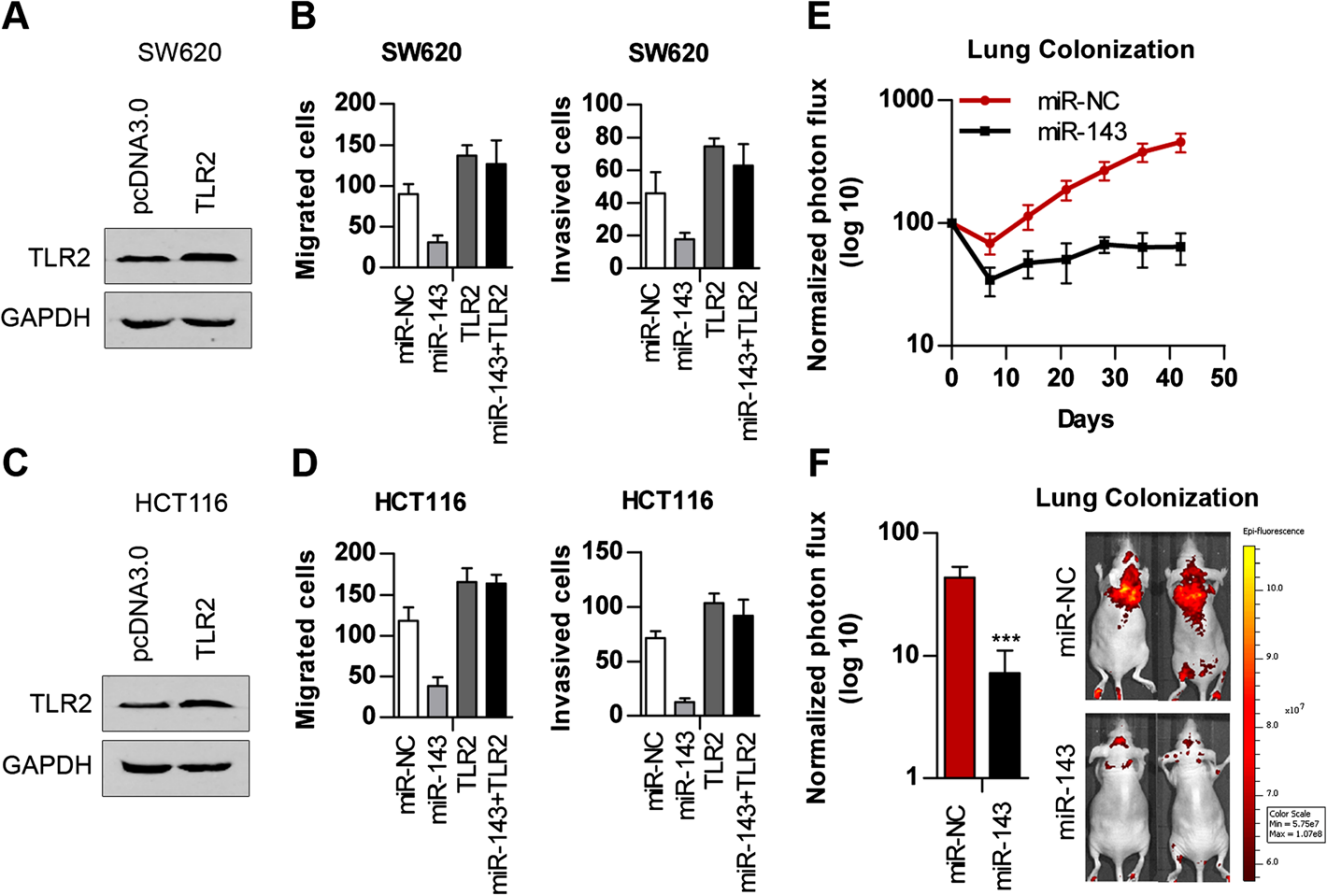
**TLR2 modulation accounts for the antimetastatic effect of miR-143.** (**A**) Western blot showing TLR2 expression in SW620 cells 48 h after transfection with the artificial TLR2 expression vector (TLR2). (**B**) The effects of miR-143, TLR2, and miR-143 combined with TLR2 on the migration and invasion of SW620 cells. (**C**) Western blot showing TLR2 translation in HCT116 cells 48 hours after transfection with TLR2. (**D**) The effects of miR-143, TLR2, and miR-143 combined with TLR2 on the migration and invasion of HCT116 cells. (**E**) Aliquots of 1 ×10^6^ stable miR-143 or miR-NC over-expressing SW620 cells were injected into immunodeficient mice and evaluated for lung colonisation capacity in tail-vein assays. Lung colonisation was measured using bioluminescence at 1, 2, 3, 4, 5 and 6 weeks after injection. (**F**) The bar graph represents 24 hour time-point measurements of the normalised photon flux from animals injected with SW620 cells. Representative images are shown. n = 5 for each group; error bars indicate SD.

To determine whether TLR2 is the critical mediator of the role of miR-143 in cellular invasion and migration, we constructed a TLR2 transcript with a 3′UTR deletion mutation (TLR2). First, we performed a western blot analysis 48 h after transfecting the TLR2 expression vector with the 3′UTR deletion mutation (TLR2) into SW620 (Figure 5A and Additional file 5: Figure S5A) and HCT116 cells (Figures 5C and Additional file 5: Figure S5B). As shown in the figures, compared to the negative control group (pcDNA3.0), ectopic expression of TLR2 significantly increased the expression of TLR2.

Expression of the artificial TLR2 transcript (TLR2) apparently promoted the invasion and migration of colorectal carcinoma cells. Importantly, re-expression of TLR2 abolished the miR-143-induced inhibition of cell invasion and migration (Figures 5B and 5D), indicating that TLR2 is a critical mediator of the anti-invasive and anti-migration effects of miR-143 in human CRC cells.

To determine the role of endogenous miR-143 in metastatic progression, 1 ×10^6^ stable miR-143 or miR-NC over-expressing SW620 cells were injected into immunodeficient mice and evaluated for lung colonisation capacity using tail-vein assays. In Figure 5E, lung colonisation was measured using bioluminescence at 1, 2, 3, 4, 5 and 6 weeks after injection. As indicated, miR-143 over-expression in SW620 cells significantly decreased lung metastatic colonisation. Additionally, lung bioluminescence was imaged 24 h post miR-143 over-expressing SW620 cells injection (Figure 5F). We detected lower photon flux intensity in SW620 cells treated with miR-143. These results may support the role of miR-143 in inhibiting the early colonisation of colorectal carcinoma cells at the lung.

## Discussion

Recently, TLR signalling pathways have been shown to be involved in tumour growth and immune evasion. Various microbial products have been used as adjuvants due to their ability to enhance tumour immunotherapy by stimulating TLR signalling and activation of both innate and adaptive immune responses [31]. However, the results of studies using TLR agonists as adjuvants in cancer treatments are contradictory and require further investigation, as the use of TLR ligands in cancer immunotherapy could turn out to be a double-edged sword [20]. Because our studies of elevated TLR2 expression in CRC do not support the use of agonists for treatment, it is important to study TLR expression in patients to better predict possible chemotherapeutic benefits.

TLR2, a unique member of the TLR family localised to the plasma membrane, recognises lipid and protein ligands and mediates both Th1 and Th2 immune re-sponses. TLR2 has been implicated in the activation of pro-inflammatory cytokines that enhance tumour invasion and migration [32]. Elevated expression of TLR2 in our cancer patient group could be associated with invasive or migratory activity. In a series of in vitro experiments, TLR-2 was identified as a critical receptor for mediating inflammation via activation of the intracellular signalling pathway that results in the translocation of NF-κB and the secretion of TNF-α and IL-1β [33]. In both tumour and host cells, TLR2 plays a crucial role in the establishment of a tumour-induced immunosuppressive environment. Targeting TLR2 leads, directly or indirectly, to the induction of strong anti-tumour immunity, which places TLR2 in a unique position as a promising target for cancer immunotherapy. However, the cause of TLR2 overexpression in human cancers has not been elucidated. The results presented here are the first line of evidence in support of the hypothesis that over-expression of TLR2 in CRC may result from under-expression of a specific miRNA molecule, miR-143; thus, our results demonstrate a new regulatory mechanism for CRC cell invasion and migration.

Aberrant miRNA expression profiles in cancer have been reported by several groups [34,35]. However, the pathological roles and molecular mechanisms of the aberrantly expressed miRNAs in CRC are poorly understood. miR-143 is particularly interesting because it possesses tumour suppressive activity and its expression is substantially reduced in several cancer types, especially in CRC [36,37]. Consistent with previous reports, our results reveal that miR-143 is under-expressed in most CRC samples. These results imply that miR-143 might serve as a novel diagnostic and therapeutic target for CRC. Many studies have identified several miR-143 targets, including MDM2 [38], KRAS [39], and HK2 [40]. Ricci-Vitiani et al. found that restoring miR-143 and miR-145 in colon cancer cells decreases proliferation, migration and chemoresistance. They also identified CD44, KLF5, and BRAF as proteins targeted by miR-143 and miR-145 [40]. As with many other miRNAs, the biological information available for miR-143 is largely limited to expression analysis. One significant obstacle that has limited the interpretation of many miRNA-profiling studies is that it is relatively difficult to identify specific miRNA targets.

In this study, overexpression of TLR2 was identified in a large-scale study of human CRC tissues. We also detected the pro-invasive and pro-migratory effects of TLR2 in CRC cell lines. The results indicate that TLR2 may function as an oncogene. To further investigate the causes of high TLR2 expression, we found a significant inverse correlation between TLR2 expression and miR-143 expression in CRC tissues using bioinformatics methods and nonparametric tests. These results indicate that the expression of TLR2 may be regulated by miR-143. For further study of the mechanisms, we used a dual luciferase report assay system to identify the functional binding site for miR-143 in TLR2 mRNA. We identified TLR2 as a novel target of miR-143 in CRC cells.

Next, we demonstrated that restoration of high miR-143 expression levels in CRC cells (SW620 and HCT116) is associated with decreased TLR2 expression. Considering the function of TLR2 in a pro-invasive and pro-migratory phenotype, we hypothesised that miR-143 may have the opposite function. As expected, miR-143 was able to reduce cancer cell invasion and migration. In animal experiments, miR-143 over-expression in SW620 cells significantly decreased lung metastatic colonisation. We demonstrated both in vivo and in vitro that miR-143 causes TLR2 down-regulation and subsequently inhibits cancer cell invasion and migration.

It was also important to note that miR-143 was more effective than siRNA (siTLR2) in inhibiting SW620 and HCT116 cell invasion and migration (Figures 2C, 2D, and 4E), despite the finding that the down-regulation of TLR2 translation by miR-143 was not more effective than siRNA (siTLR2) (Figures 2A and 4A). These findings suggest that other miR-143 targets may also play an important role in inhibiting cellular invasion and migration in CRC cells, such as DNMT3A [41], CD44, KLF5, KRAS and BRAF [40]. In addition, restoring miR-143 in colon cancer cells could inhibit proliferation [40]. However, TLR2 knockdown did not affect the growth of colorectal carcinoma cells (Figure 2B). These findings suggest that TLR2 is a novel member of numerous targets for miR-143 and that different targets can mediate the specific effects of miR-143.

In summary, our results show that miR-143 and TLR2 form a novel regulatory pathway that controls CRC cell invasion and migration. Deciphering this mechanism is an important step towards unravelling the regulatory network that underlies tumourigenesis, thereby helping us realise the potential of miRNA in cancer treatment. Additionally, using TLR agonists as adjuvants in cancer treatment appears contradictory and requires further investigation.

## Competing interests

The authors declare that they have no competing interests.

## Authors’ contributions

Haiyan Guo carried out the molecular biology studies and drafted the manuscript. Ying Chen carried out the bioinformatic analysis. Xiaobo Hu participated in the cell migration and invasion assays. Guanxiang Qian carried out the immunohistochemistry analysis. Shengfang Ge participated in the design of the study and performed the statistical analysis. Jianjun Zhang participated in the design of the study and coordination and helped to draft the manuscript. All authors read and approved the final manuscript.

## Supplementary Material

Additional file 1: Figure S1 TLR2 is highly expressed in human CRC tissues. Click here for file

Additional file 2: Figure S2 TLR2 mediates the capacity for invasion and migration in CRC cells. Click here for file

Additional file 3: Figure S3 Expression of miR-143 in normal colon epithelial cells and CRC cells. Click here for file

Additional file 4: Figure S4 miR-143 directly targets TLR2. Click here for file

Additional file 5: Figure S5 Expression of TLR2 in CRC cells treated with the TLR2 expression vector. Click here for file
