# Supplementary figures and images for: The regulation of toll-like receptor 2 by miR-143 suppresses the invasion and migration of a subset of human colorectal carcinoma cells
Source: Mol Cancer. 2013 Jul 17;12:77. doi: 10.1186/1476-4598-12-77 (PMC3750391; doi:10.1186/1476-4598-12-77)

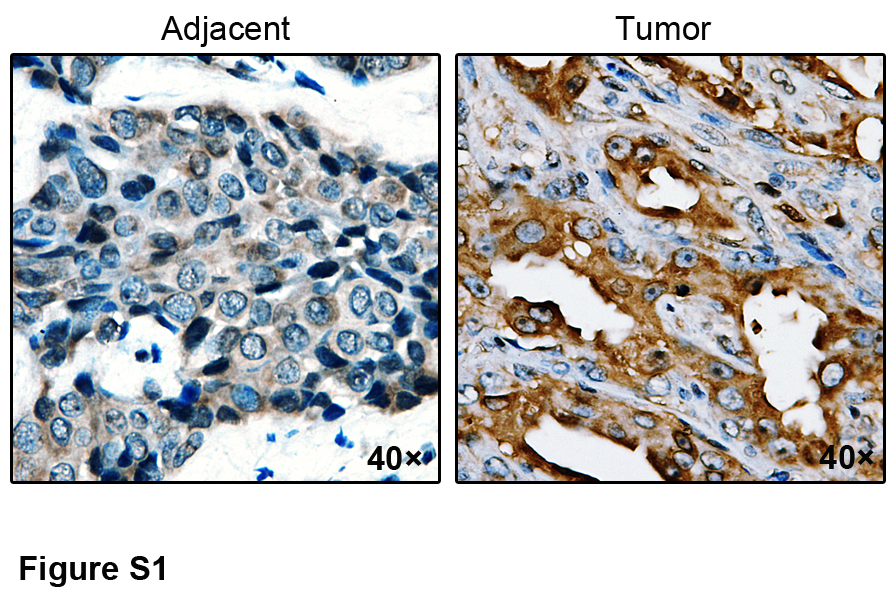

Supplement: Additional file 1: Figure S1 — TLR2 is highly expressed in human CRC tissues. [file 1476-4598-12-77-S1.tiff]

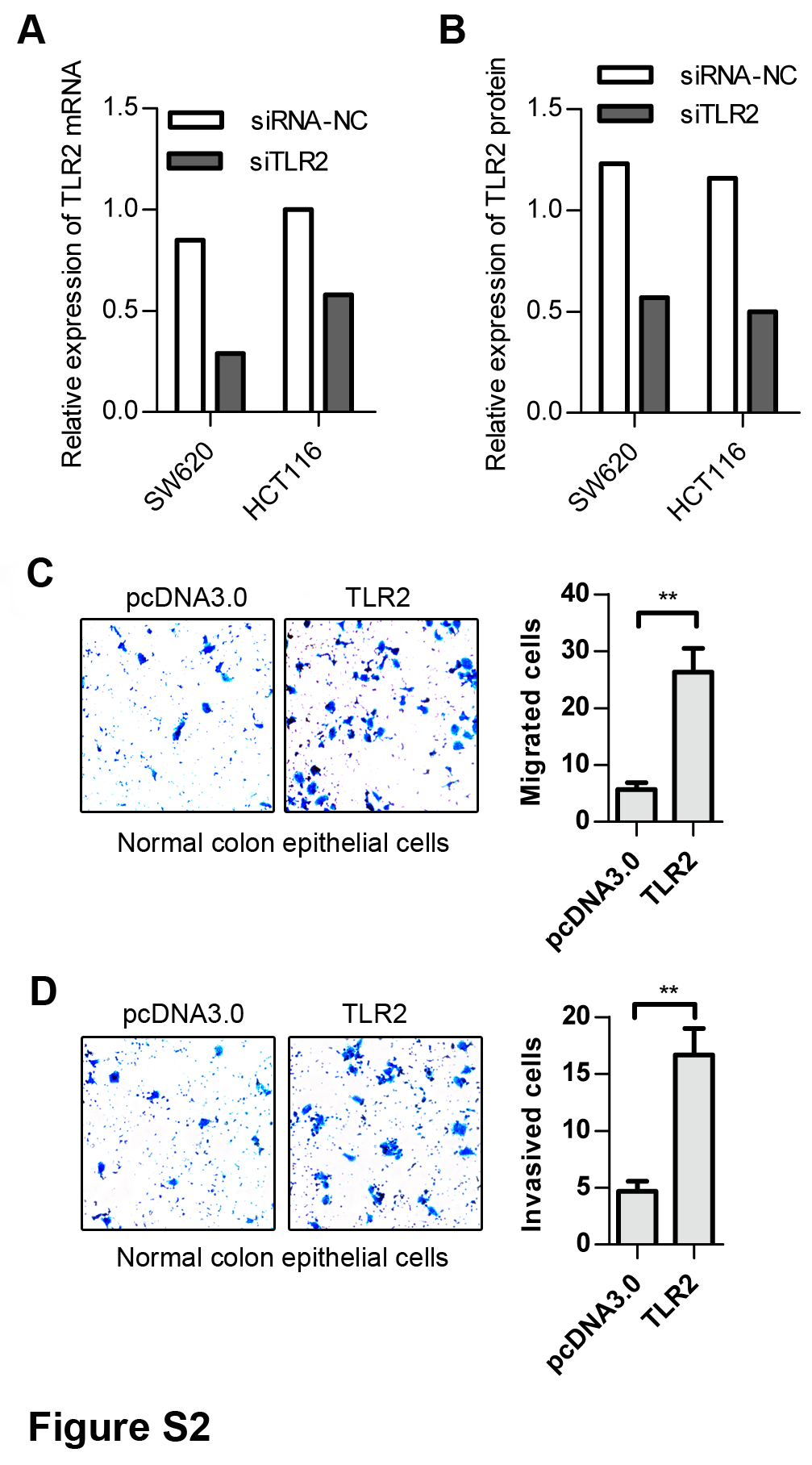

Supplement: Additional file 2: Figure S2 — TLR2 mediates the capacity for invasion and migration in CRC cells. [file 1476-4598-12-77-S2.tiff]

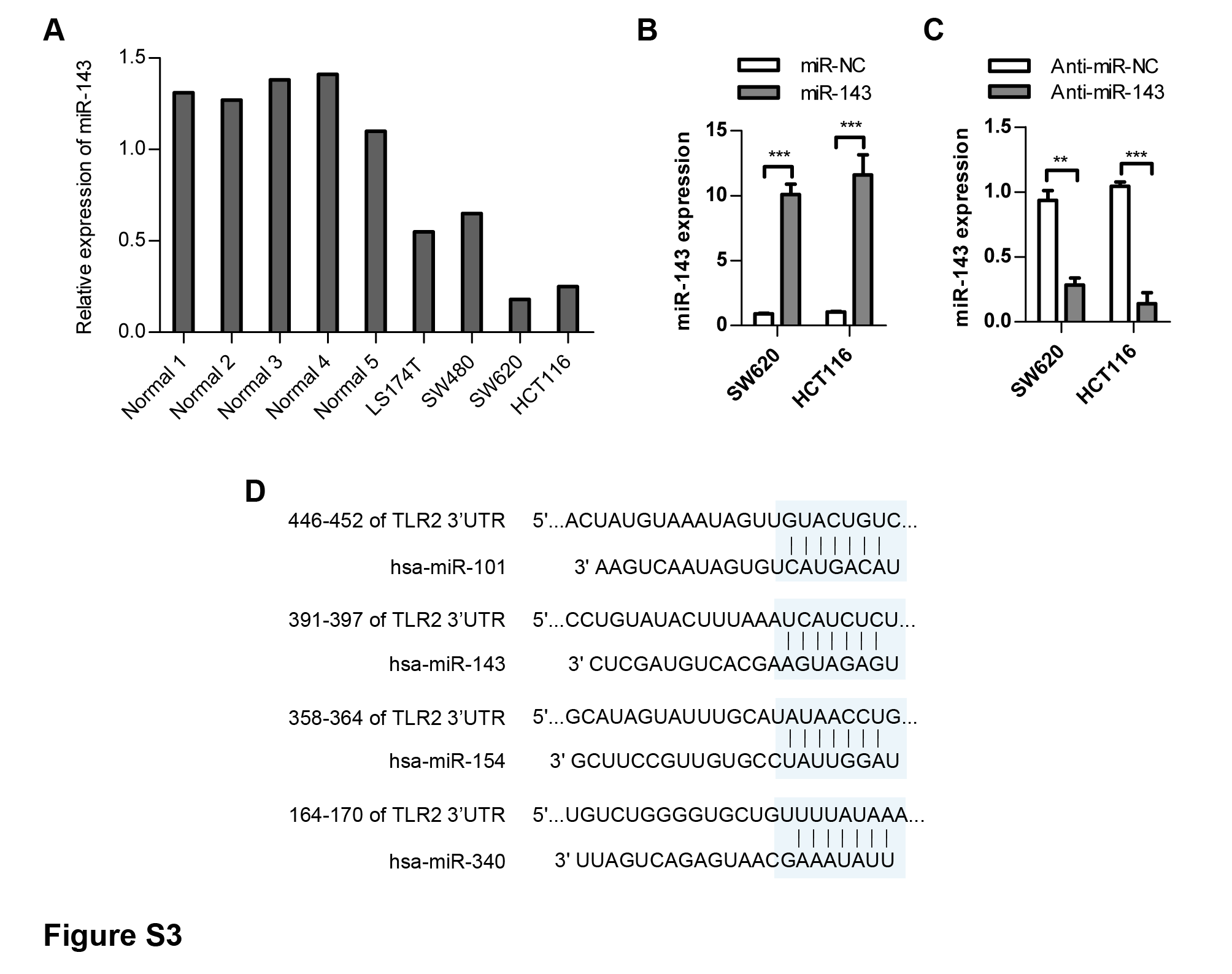

Supplement: Additional file 3: Figure S3 — Expression of miR-143 in normal colon epithelial cells and CRC cells. [file 1476-4598-12-77-S3.tif]

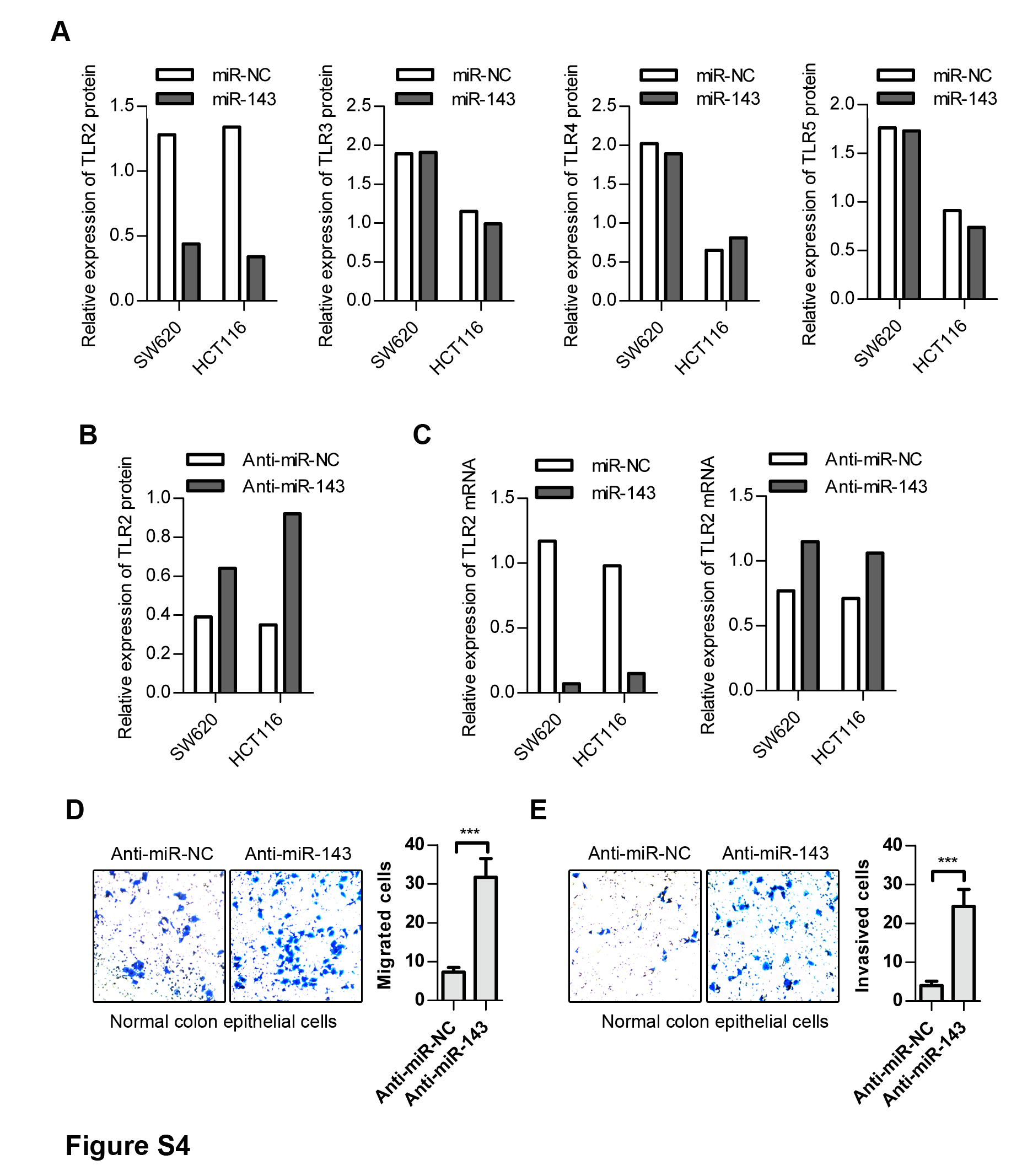

Supplement: Additional file 4: Figure S4 — miR-143 directly targets TLR2. [file 1476-4598-12-77-S4.tiff]

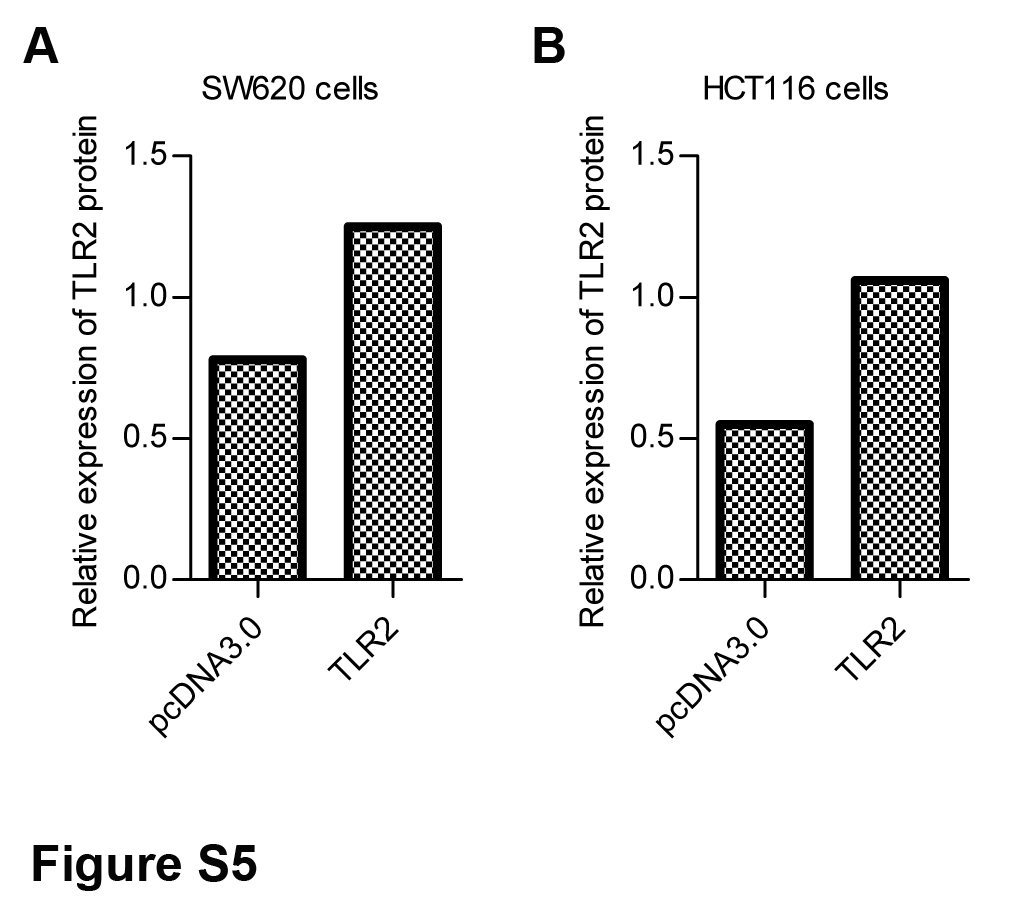

Supplement: Additional file 5: Figure S5 — Expression of TLR2 in CRC cells treated with the TLR2 expression vector. [file 1476-4598-12-77-S5.tiff]
